# Prescription of Potentially Inappropriate Medication Use in Older Cancer Outpatients With Multimorbidity: Concordance Among the Chinese, AGS/Beers, and STOPP Criteria

**DOI:** 10.3389/fphar.2022.857811

**Published:** 2022-04-12

**Authors:** Fangyuan Tian, Mengnan Zhao, Zhaoyan Chen, Ruonan Yang

**Affiliations:** ^1^ Department of Pharmacy, West China Hospital, Sichuan University, Chengdu, China; ^2^ Department of Epidemiology and Health Statistics, West China School of Public Health and West China Fourth Hospital, Sichuan University, Chengdu, China

**Keywords:** potentially inappropriate medications, cancer, older, criteria, outpatient

## Abstract

**Objectives:** Age-related multimorbidity is a general problem in older patients, which increases the prevalence of potentially inappropriate medication (PIM) use. This study aimed to examine the prevalence and predictors of PIM use in older Chinese cancer outpatients with multimorbidity based on the 2017 Chinese criteria, 2019 AGS/Beers criteria, and 2014 STOPP criteria.

**Methods:** A cross-sectional study was conducted using electronic medical data from nine tertiary hospitals in Chengdu from January 2018 to December 2018. The 2017 Chinese criteria, 2019 AGS/Beers criteria, and 2014 STOPP criteria were used to evaluate the PIM status of older cancer outpatients (age ≥65 years), the concordance among the three PIM criteria was calculated using kappa tests, and multivariate logistic regression was used to identify the risk factors associated with PIM use.

**Results:** A total of 6,160 cancer outpatient prescriptions were included in the study. The prevalence of PIM use was 34.37, 32.65, and 15.96%, according to the 2017 Chinese criteria, 2019 AGS/Beers criteria, and 2014 STOPP criteria, respectively. Furthermore, 62.43% of PIMs met table 2, 0.27% of PIMs met table 3, 34.68% of PIMs met table 4, 2.62% of PIMs met table 5 of 2019 AGS/Beers criteria, respectively. According to the three criteria, 84.93%, 82.25%, and 94.61% of older cancer outpatients had one PIM. The most frequently used PIM in cancer outpatients was estazolam. The Chinese criteria and the STOPP criteria indicated poor concordance, whereas the 2019 AGS/Beers criteria showed moderate concordance with the other two criteria. Logistic regression demonstrated that age ≥ 80, more diseases, polypharmacy, irrational use of drugs, and lung cancer were positively associated with PIM use in older cancer outpatients.

**Conclusion:** The prevalence of PIM use in Chinese older cancer outpatients with multimorbidity is high in China, and poor-to-moderate concordance among the three criteria was observed. Research on building PIM criteria for the older cancer population is necessary in the future.

## Introduction

With the global population aging, the total number of people aged 60 years and older in the world is expected to reach 2 billion by 2050. China is the most populous country in the world, and the older population is also the largest (Jia et al., 2020). Older adults are more likely to suffer from multiple diseases, especially chronic diseases requiring complex treatments, such as taking many different medicines (Cojutti et al., 2016). Polypharmacy (defined as more than five medicines) is associated with the prescription of inappropriate medications, and a growing body of evidence links polypharmacy with negative outcomes (Field et al., 2001; Ferner and Aronson, 2006; Maddison et al., 2011; Weng et al., 2013; LeBlanc et al., 2015).

However, alterations in age-related pharmacokinetics and pharmacodynamics of older adults have led to an increased risk of drug–drug interactions and drug–disease interactions (Fried et al., 2014; Payne, 2016). Cancer patients are particularly prone to unintended consequences of polypharmacy because chemotherapy may carry a risk of drug–drug interactions and adverse drug events, which may include chemotherapy-related toxicity (Maggiore et al., 2014; Woopen et al., 2016). Some studies have shown that older cancer patients could suffer from a higher rate of comorbidity, frailty, and geriatric syndrome, putting them at high risk of polypharmacy and inappropriate medication use (Wildiers et al., 2014; Koczwara et al., 2022).

Potentially inappropriate medication (PIM) is a public health issue that can be defined as medications that should be avoided and may outweigh the expected clinical benefit, such as adverse drug events, hospitalization, disability, and economic burden (Hyttinen et al., 2016; Muhlack et al., 2017; Wallace et al., 2017). The American Geriatrics Society (AGS)/Beers criteria were the first expert consensus on geriatric PIM (Beers et al., 1991). The AGS, through an expert US-based panel, has undertaken the task of regular review and updating of AGS/Beers criteria, which are now in their sixth iteration (American Geriatrics Society Beers Criteria® Update Expert Panel, 2019). There were some substantial changes in the categories, and some medications were dropped or added. Because Beers criteria were not organized according to physiological systems, University College Cork organized experts from many disciplines to formulate the screening tool of old persons’ prescriptions to alert to the right treatment (STOPP/START criteria) through the Delphi method, and the second edition was updated in 2014 (O'Mahony et al., 2015; O'Mahony, 2020). Two criteria have been widely used in PIM use application surveys in communities, clinics, and hospitals worldwide. China formulated the criteria for judging the potentially inappropriate medication use of older adults by an expert panel in 2017, including medication risk and medication risk under disease status (Rational Drug Use Branch of Chinese Association of Geriatric, 2018). These country-specific criteria were divided into high-risk and low-risk medications according to experts’ evaluation.

Some previous reports examined PIM use in older Chinese patients based on the three criteria. However, no study has specifically reported on the concordance among the three criteria. The prevalence and the risk factors associated with PIM use according to the three criteria in older Chinese cancer patients are unclear. The concordance of different criteria often led to large differences in the results. Besides, country-specific and non-country-specific criteria significantly impact PIMs in older cancer patients. Therefore, in this study, we extracted prescriptions of cancer outpatients treated at tertiary hospitals in Chengdu, China. PIMs were screened based on the 2017 Chinese, 2019 AGS/Beers, and 2014 STOPP criteria. The concordance among the three PIM criteria was calculated, and the prevalence and the risk factors associated with PIMs were explored. It is hoped that this study will provide relevant evidence for follow-up research.

## Methods

### Setting and Sample

The cross-sectional study was performed to examine the concordance between the 2017 Chinese, 2019 AGS/Beers, and 2014 STOPP criteria on the detection of PIM use among older cancer outpatients with multimorbidity in tertiary hospitals in Chengdu, a capital city in southwest China, which covers an area of 12,390 square kilometers, with a permanent population of 16.0 million in 2017. The prescriptions of older (aged ≥65) cancer outpatients with multimorbidity (cancer with other diseases) were cluster sampled from a hospital prescription analysis cooperation project led by the Chinese Pharmaceutical Association between 1 January and 31 December 2018. All data were retrospectively encoded without any possibility of identification and treated.

### Data Collection

The data were collected by diagnoses type as follows: 1) basic information (region, prescription number, and department source); 2) patient characteristics (age, gender, and diagnosis); and 3) medication characteristics (generic name, trade name, specification, dosage form, administration route, number of prescriptions, prescription expenditure dosage, and frequency of administration).

### Evaluation Criteria

The 2017 Chinese, 2019 AGS/Beers, and 2014 STOPP criteria were used to evaluate PIM use for older cancer outpatients outside of palliative care and hospice service. The prescription in this study was evaluated as potentially inappropriate with PIM use in older adults (table 2), PIM use in older adults due to drug-disease or drug-syndrome interactions that may exacerbate the disease or syndrome (table 3, drugs to be used with caution in older adults (table 4, and potentially clinically important drug–drug interactions that should be avoided in older adults (table 5 of 2019 AGS/Beers Criteria. The 2014 STOPP criteria were used (not including a screening tool to alert to right treatment criteria). The 2017 Chinese criteria contained two tables about PIM use in Chinese older adults and PIM use in Chinese older adults under disease states. PIM was divided into high-risk and low-risk medications and divided into A and B categories according to defined daily doses. Researchers (FY Tian, RN Yang) independently reviewed the medications of each patient and assessed prescription expenditure. Prescription expenditure refers to the expenditure of all drugs in the prescription. The irrational use of the drugs was evaluated by two clinical pharmacists (FY Tian, ZY Chen). Prescription comments were done according to the Chinese Prescription Administrative Policy. Nonstandard prescriptions, inappropriate prescriptions, and supernormal prescriptions referring to medication without indications were classified as irrational prescriptions. Any inconsistencies between the two researchers were submitted to a third professional and then resolved through collective discussion.

### Statistical Analysis

Categorical data were described using frequency, and the χ^2^ test was used to compare categorical variables between groups. Continuous data subject to a normal distribution are expressed as the mean ± standard deviation (SD), and continuous data subject to a nonnormal distribution are expressed as M (P_25_, P_75_). We defined gender, age, number of diseases, polypharmacy, rational prescriptions, expenditure, and type of cancer as risk factors. The associations between risk factors and PIM use (non-PIM = 0, PIM = 1) were performed through multivariate logistic regression analysis to determine the influence on PIM-related risk. Statistical analyses were conducted using SPSS version 26.0 (Armonk, NY: IBM Corp.). A comparative analysis was performed between the results obtained for the three PIM identification tools, and the agreement between them was determined through weighted kappa concordance tests (values of kappa >0.60 indicate good to excellent agreement, values between 0.40 and 0.60 indicate moderate agreement, and values < 0.40 indicate poor agreement) (Landis and Koch, 1977). Logistic regression used the enter method strategy and likelihood ratio method. The results of the logistic regression analysis are presented with odds ratios (ORs) and 95% confidence intervals (CIs); *p* < 0.05 was considered statistically significant.

### Ethics Approval

This study protocol was approved by the Sichuan University West China Hospital Research Ethics Board. All procedures performed in this study conformed to the standards of the 1964 Helsinki Declaration and subsequent relevant ethics.

## Results

### Basic Characteristics of Patients

A total of 6,160 cancer outpatient prescriptions were included in this study, of which 46.53% (2,866) were female. The median age was 72 (IQR: 68, 78) years old, ranging from 65 to 99, with the oldest (≥80 years of age) cancer patients accounting for 18.72% (1,153). The median number of medical diagnoses was 3 (IQR: 2, 5). Regarding medication of prescriptions, the median number prescribed was 3 (IQR: 2, 4), and 22.53% (1,388) of older cancer outpatients had polypharmacy. The prevalence of rational prescriptions was 93.93% (5,786). The median prescription expenditure was 814.62 (IQR: 274.65, 1,638.95) Chinese Yuan (CNY). In this study, 20.70% (1,275) of the patients had lung cancer, 18.83% (1,160) had breast cancer, 16.36% (1,008) had colorectal cancer, 12.76% (786) had prostate cancer, and 6.38% (393) had gastric cancer The characteristics of the basic information in this study are listed in Table 1.

**TABLE 1:** Basic characteristics of older cancer outpatients.

Characteristics	Total	2017 Chinese criteria			2019 AGS/Beers criteria			2014 STOPP criteria		
		PIM group	Non-PIM group	*p*-value	PIM group	Non-PIM group	*p*-value	PIM group	Non-PIM group	*p*-value
N (%)	6,160	2,117 (34.37)	4,043 (65.63)		2011 (32.65)	4,149 (63.35)		983 (15.96)	5,177 (84.04)	
Sex, n (%)				<0.001			0.023			0.016
Male	3,294 (53.47)	1,228 (58.01)	2066 (51.10)		1,117 (55.54)	2,177 (52.47)		491 (49.95)	2,803 (54.14)	
Female	2,866 (46.53)	889 (41.99)	1977 (48.90)		894 (44.46)	1972 (47.53)		482 (49.03)	2,374 (45.86)	
Age, years (IQR), n (%)	72 (68, 78)			<0.001			<0.001	743 (75.58)	4,264 (82.36)	<0.001
65–79	5,007 (81.28)	1,631 (77.04)	3,376 (83.50)		1,580 (78.57)	3,427 (82.60)		240 (24.42)	913 (17.64)	
≥80	1,153 (18.72)	486 (22.96)	667 (16.50)		431 (21.43)	722 (17.40)				
No. of diseases (IQR)	3 [2, 5]			<0.001			0.002			<0.001
2	1941 (31.51)	567 (25.58)	1,374 (33.98)		581 (28.89)	1,360 (32.78)		210 (21.36)	1731 (33.44)	
3-4	2,619 (42.52)	897 (42.37)	1722 (42.59)		860 (42.76)	1759 (42.40)		402 (40.90)	2,217 (42.82)	
≥5	1,600 (25.97)	653 (30.85)	947 (23.42)		570 (28.34)	1,030 (24.83)		371 (37.74)	1,229 (23.74)	
No. of medications (IQR), n (%)	3 [2, 4]			<0.001			<0.001			<0.001
1–4	4,772 (77.47)	1,391 (65.71)	3,381 (83.63)		1,400 (69.61)	3,372 (81.27)		615 (62.56)	4,157 (80.30)	
≥5	1,388 (22.53)	726 (34.29)	662 (16.37)		611 (30.38)	777 (18.73)		368 (37.44)	1,020 (19.70)	
No. of rational prescriptions, n (%)				<0.001			<0.001			<0.001
Rational prescriptions	5,786 (93.93)	1928 (91.07)	3,858 (95.42)		1828 (90.90)	3,958 (95.40)		859 (87.39)	4,927 (95.17)	
Irrational prescriptions	374 (6.07)	189 (8.93)	185 (4.58)		183 (9.10)	191 (4.60)		124 (12.61)	250 (4.83)	
No. of prescription expenditures [(QR), n (%)	814.62 (274.65, 1,639.95)									0.813
<500 CNY	2,322 (37.69)	874 (41.28)	1,448 (35.81)	<0.001	939 (46.69)	1,383 (33.33)	<0.001	378 (38.45)	1944 (37.55)	
500–1000 CNY	1,108 (17.99)	376 (17.76)	732 (18.11)		321 (15.96)	787 (18.97)		171 (17.40)	937 (18.10)	
>1000 CNY	2,730 (44.32)	867 (40.95)	1863 (46.08)		751 (37.34)	1979 (47.40)		434 (44.15)	2,296 (44.35)	
Type of chronic disease, n (%)										
Lung cancer	1,275 (20.70)	554 (26.17)	721 (17.83)	<0.001	544 (27.05)	731 (17.62)	<0.001	264 (26.86)	1,011 (19.53)	<0.001
Breast cancer	1,160 (18.83)	251 (11.86)	909 (22.48)	<0.001	253 (12.58)	907 (21.86)	<0.001	157 (15.97)	1,003 (19.37)	0.012
Colorectal cancer	1,008 (16.36)	376 (17.76)	632 (15.63)	0.032	384 (19.09)	624 (15.04)	<0.001	184 (18.72)	824 (15.92)	0.03
Prostate cancer	786 (12.76)	315 (14.88)	471 (11.65)	<0.001	247 (12.28)	539 (12.99)	0.434	132 (13.43)	654 (12.63)	0.493
Gastric cancer	393 (6.38)	114 (5.38)	279 (6.90)	0.021	114 (5.67)	279 (6.72)	0.112	56 (5.70)	337 (6.51)	0.339
Liver cancer	379 (6.15)	109 (5.15)	270 (6.68)	0.018	99 (4.92)	280 (6.75)	0.005	30 (3.05)	349 (6.74)	<0.001
Esophageal cancer	298 (4.84)	74 (3.50)	224 (5.54)	<0.001	74 (3.68)	224 (5.40)	0.003	15 (1.53)	283 (5.47)	<0.001
Uterine cancer	130 (2.11)	35 (1.65)	95 (2.35)	0.071	37 (1.84)	93 (2.24)	0.304	21 (2.14)	109 (2.11)	0.951
Kidney Cancer	125 (2.03)	49 (2.31)	76 (1.88)	0.25	43 (2.14)	82 (1.98)	0.673	23 (2.34)	102 (1.97)	0.451
Thyroid cancer	117 (1.90)	35 (1.65)	82 (2.03)	0.306	27 (1.34)	90 (2.17)	0.026	21 (2.14)	96 (1.85)	0.553

PIM, potentially inappropriate medication; IQR, interquartile range; CNY, Chinese yuan.

### Concordance Between the Three Criteria

Considering the three PIM classification tools applied, the 2017 Chinese criteria had 1335 PIM prescriptions in common with the 2019 AGS/Beers criteria and 726 PIM prescriptions in common with the 2014 STOPP criteria. In contrast, the 2019 AGS/Beers criteria had 919 PIMs in common with the 2014 STOPP criteria. The kappa statistic for the 2017 Chinese and STOPP criteria was 0.320, indicating poor concordance. In contrast, the 2019 AGS/Beers criteria showed moderate concordance with the 2017 Chinese criteria and the 2014 STOPP criteria (*κ* = 0.469 and 0.509, respectively) (Table 2).

**TABLE 2:** Concordance between the 2017 Chinese, 2019 AGS/Beers, and 2014 STOPP criteria.

2019 AGS/Beers criteria	2017 Chinese criteria		κ	P
	Yes	No		
Yes	1,335	676	0.469	<0.001
No	782	3,367		
**2014 STOPP criteria**	**2017 Chinese criteria**			
	Yes	No		
Yes	726	257	0.320	<0.001
no	1,391	3,786		
**2019 AGS/Beers criteria**	**2014 STOPP criteria**			
	Yes	No		
Yes	919	1,092	0.509	<0.001
No	64	4,085		

κ, kappa coefficient; P, probability value, based on kappa test.

### Prevalence of PIMs and the Most Frequent PIMs

Among the 6,160 older cancer outpatient prescriptions, 2,117 (34.37%) outpatient prescriptions were identified with at least one PIM, and a total of 2,477 PIMs were detected by the 2017 Chinese criteria. Of the patient prescriptions with PIM, 84.93% received one PIM, 13.04% received two PIMs, and 2.03% had at least three PIMs according to the criteria (Table 3). Overall, the most consumed PIMs according to the 2017 Chinese criteria were estazolam, clopidogrel, and tramadol at 20.65%, 14.00%, 13.68%, respectively (Table 4).

**TABLE 3:** The number of PIMs used by older cancer outpatients in the PIM group.

Characteristics	2017 Chinese criteria	2019 AGS/Beers criteria	2014 STOPP criteria
PIM prescription	2,117	2011	983
PIMs, n (%)	2,477	2,630	1,036
1 PIM	1798 (84.93)	1,654 (82.25)	930 (94.61)
2 PIMs	276 (13.04)	215 (10.69)	41 (4.17)
≥3 PIMs	43 (2.03)	142 (7.06)	12 (1.22)

PIM, potentially inappropriate medication.

**TABLE 4:** The five most consumed PIMs used by older cancer outpatients.

Number	2017 Chinese criteria	N = 2,464 (%)	2019 AGS/Beers criteria	N = 2,427 (%)	2014 STOPP criteria	N = 1,022 (%)
1	Estazolam	509 (20.65)	Estazolam	509 (20.97)	Estazolam	509 (49.80)
2	Clopidogrel	345 (14.00)	Tramadol	337 (13.89)	Glimepiride	180 (17.61)
3	Tramadol	337 (13.68)	Hydrochlorothiazide	239 (9.85)	Alprazolam	161 (15.75)
4	Mixed insulin	201 (8.16)	Glimepiride	180 (7.42)	Zolpidem	29 (2.84)
5	Insulin glargine	189 (7.67)	Alprazolam	161 (6.63)	Flupentixol and melitracen	24 (2.35)

According to the 2019 AGS/Beers criteria, 2011 (32.65%) outpatient prescriptions were identified with at least one PIM, and a total of 2,630 PIMs were detected. Among them, 62.43% met table 2, 0.27% met table 3, 34.68% met table 4, and 2.62% met table 5 of 2019 AGS/Beers criteria, respectively. Of the patient prescriptions with PIM, 82.25% received one PIM, 10.69% received two PIMs, and 7.06% had at least three PIMs according to the criteria (Table 3). Overall, the most consumed PIMs according to the 2019 AGS/Beers criteria were estazolam, tramadol, and hydrochlorothiazide, which were 20.97%, 13.89%, 9.85%, respectively (Table 4).

Based on the 2014 STOPP criteria, 983 (15.96%) outpatient prescriptions were identified with at least one PIM, and 1,036 PIMs were detected. Of the patient prescriptions with PIM, 94.61% received one PIM, 4.17% received two PIMs, and 1.22% were had at least three PIMs according to the criteria (Table 3). Overall, the most consumed PIMs according to the 2014 STOPP criteria were estazolam, glimepiride, and alprazolam at 49.80%, 17.61%, and 15.75%, respectively (Table 4).

### Risk Factors for PIM Use

Based on the three criteria, PIM use was the dependent variable (non-PIM = 0, PIM = 1). Logistic regression demonstrated that age ≥ 80 years (OR: 1.322 by 2017 Chinese criteria, OR: 1.238 by 2019 AGS/Beers criteria, OR: 1.386 by 2014 STOPP criteria), more diseases (OR: 1.348 by 2017 Chinese criteria, OR: 1.193 by 2019 AGS/Beers criteria, OR: 2.229 by 2014 STOPP criteria), polypharmacy (OR: 3.09 by 2017 Chinese criteria, OR: 2.52 by 2019 AGS/Beers criteria, OR: 2.087 by 2014 STOPP criteria), and irrational use of drugs (OR: 1.679 by 2017 Chinese criteria, OR: 1.762 by 2019 AGS/Beers criteria, OR: 2.857 by 2014 STOPP criteria) were positively associated with PIM use in older cancer outpatients. Lung cancer patients (OR: 1.281 by 2017 Chinese criteria, OR: 1.344 by 2019 AGS/Beers criteria, OR: 1.421 by 2014 STOPP criteria) were also more likely to have PIMs. However, when the prescription expenditure (OR: 0.524 by 2017 Chinese criteria, OR: 0.416 by 2019 AGS/Beers criteria, OR: 0.634 by 2014 STOPP criteria) was higher, PIM use in older cancer outpatients was lower (Table 5).

**TABLE 5:** Multivariate logistic regression analysis of factors associated with PIM use.

	2017 Chinese criteria 2				2019 AGS/Beers criteria				2014 STOPP criteria		
Characteristics	OR	95% CI	*p*-value	Characteristics	OR	95% CI	*p*-value	Characteristics	OR	95% CI	*p*-value
Sex				Sex				Sex			
Female	References		Female	References		Female	References				
Male	1.026	0.895–1.176	0.714	Male	0.934	0.814–1.073	0.337	Male	0.754	0.634–0.897	0.001
Age				Age				Age			
65–79	References		65–79	References		65–79	References				
≥80	1.322	1.146–1.525	<0.001	≥80	1.238	1.071–1.431	0.004	≥80	1.386	1.164–1.652	<0.001
No. of diseases				No. of diseases				No. of diseases			
2	References		2	References		2	References				
3-4	1.205	1.053–1.380	0.007	3-4	1.157	1.157–1.193	0.035	3-4	1.5	1.244–1.815	<0.001
≥5	1.348	1.152–1.579	<0.001	≥5	1.193	1.017–1.399	0.03	≥5	2.229	1.815–2.737	<0.001
No. of medications				No. of medications				No. of medications			
1–4	References		1–4	References		1–4	References				
≥5	3.09	2.667–3.58	<0.001	≥5	2.52	2.169–2.927	<0.001	≥5	2.087	1.747–2.493	<0.001
No. of rational prescriptions				No. of rational prescriptions				No. of rational prescriptions			
Rational prescriptions	References		rational prescriptions	References		rational prescriptions	References				
Irrational prescriptions	1.679	1.339–2.104	<0.001	irrational prescriptions	1.762	1.408–2.205	<0.001	irrational prescriptions	2.857	2.233–3.657	<0.001
No. of prescription expenditures				No. of prescription expenditures				No. of prescription expenditures			
<500 CNY	References		<500 CNY	References		<500 CNY	References				
500–1000 CNY	0.665	0.566–0.782	<0.001	500–1000 CNY	0.488	0.414–0.576	<0.001	500–1000 CNY	0.714	0.578–0.882	0.002
>1000 CNY	0.524	0.454–0.604	<0.001	>1000 CNY	0.416	0.360–0.480	<0.001	>1000 CNY	0.634	0.527–0.7630	<0.001
Type of chronic disease				Type of chronic disease				Type of chronic disease			
Lung cancer	1.281	1.067–1.538	0.008	Lung cancer	1.344	1.066–1.694	0.013	Lung cancer	1.421	1.125–1.794	0.003
Breast cancer	0.514	0.412–0.640	<0.001	Breast cancer	0.598	0.462–0.776	<0.001	Breast cancer	-	-	0.084
Colorectal cancer	—	—	0.243	Colorectal cancer	—	—	0.545	Colorectal cancer	—	—	0.557
Prostate cancer	—	—	0.346	Prostate cancer	—	—	0.876	Prostate cancer	—	—	0.298
Gastric cancer	0.62	0.476–0.808	<0.001	Gastric cancer	0.721	0.535–0.970	0.031	Gastric cancer	—	—	0.469
Liver cancer	0.757	0.577–0.993	0.044	Liver cancer	—	—	0.072	Liver cancer	0.54	0.354–0.825	0.004
Esophageal cancer	0.542	0.399–0.736	<0.001	Esophageal cancer	0.57	0.407–0.798	0.001	Esophageal cancer	0.31	0.177–0.7542	<0.001
Uterine cancer	0.631	0.411–0.969	0.035	Uterine cancer	—	—	0.126	Uterine cancer	—	—	0.599
Kidney cancer	—	—	0.392	Kidney cancer	—	—	0.392	Kidney cancer	—	—	0.871
Thyroid cancer	0.624	0.407–0.958	0.031	Thyroid cancer	0.48	0.299–0.771	0.002	Thyroid cancer	—	—	0.988

## Discussion

To the best of our knowledge, this is the first study assessing the concordance of three PIM-detecting tools—the 2017 Chinese criteria, the 2019 AGS/Beers criteria, and the 2014 STOPP criteria—in older Chinese cancer outpatients. Although these criteria were developed for different populations and with different aims, they are the most commonly used in older Chinese patients. Because multiple comorbidities are frequent among older cancer patients, a tool focusing on cancer outpatients should be implemented to alert doctors to an eventual PIM prescription. Our study found that the 2017 Chinese and the 2014 STOPP criteria indicated poor coherence, whereas the 2019 AGS/Beers criteria showed moderate concordance with the other two criteria, which was a little different from another study on Chinese older inpatients (Ma et al., 2018). Moreover, a Portuguese study performed in inpatients 65 or more years of age showed poor concordance among the 2019 AGS/Beers criteria, 2014 STOPP criteria, and the EU(7)-PIM list (Perpétuo., 2021). The low concordance between different criteria highlights the need to develop special PIM-detecting criteria for older cancer patients exposed to many PIMs and reinforces the fact that older cancer outpatients are also at risk of PIM. This will provide a basis for rational drug use for cancer patients and reduce outpatient prescription expenditure. The poor concordance between the Chinese and the STOPP criteria can be due to the applicability requirements of each list. The overlap between the Beers criteria and the other two criteria regarding medication risk irrespective of conditions was relatively high. However, the Chinese criteria contained clopidogrel and mixed insulin not included in the Beers criteria. In order to determine one PIM with the STOPP criteria, it is imperative to know the entire medication history and clinical information of the patient. These reasons may lead to moderate concordance between the Beers criteria and the other two criteria.

China is currently the country with the largest older cancer population in the world, and cancer as a chronic disease places a heavy burden on the elderly. Older cancer patients can suffer from a higher rate of comorbidity, frailty, and geriatric syndrome, putting them at a high risk of polypharmacy and PIM use (Pamoukdjian et al., 2020; Kleckner et al., 2022). To the best of our knowledge, this is the first cross-sectional study on the prevalence and risk factors for PIM use in Chinese older cancer outpatients according to the three criteria. The prevalence of PIM use was 34.37%, 32.65%, and 15.96%, according to the 2017 Chinese, 2019 AGS/Beers, and 2014 STOPP criteria, respectively. There is little difference between the 2017 Chinese and 2019 AGS/Beers criteria. However, the prevalence of PIM use of the 2014 STOPP criteria was lower than the other two criteria. According to the 2017 Chinese criteria, to consider the medicine as a PIM, it is only necessary to know the status of medication and disease in older patients. In addition, Chinese criteria were made based on drug utilization of the older Chinese population, so it is more suitable for Chinese individuals. The AGS/Beers criteria judge each medicine as a PIM based not only on the medication profile of a patient but also on the pathologies of the patients, as well as the laboratory results (O'Mahony et al., 2015). In order to apply the STOPP criteria, it is imperative to know the entire medication history, clinical information of the patient, and laboratory (O'Mahony et al., 2015; O'Mahony, 2020; Perpétuo et al., 2021). Based on the 2019 AGS/Beers criteria, our study found that the prevalence of PIM use among older Chinese cancer patients was 32.65%, which was lower than the prevalence of 80.4% reported by a study on Korean cancer patients according to the 2019 AGS/Beers criteria (Suh et al., 2021). The older Korean patients received anti-neoplastic therapy with emergency department (ED) visits, the prevalence of polypharmacy in the patients was observed in 80.4%, and the prevalence was 22.53% in our study. Taking more medications was the reason for the higher prevalence of PIM use compared to our study. Based on the 2014 STOPP criteria, our study found that the prevalence of PIM use among older cancer outpatients was 15.96%, which was lower than Japanese with a prevalence of 31.9% (Hakozaki et al., 2021). Older advanced non–small cell lung cancer (NSCLC) patients and those on oral molecular-targeted anticancer agents were included in the study. According to our research, the prevalence of PIM use in lung cancer patients was higher. The high prevalence of PIM use is that older cancer outpatients are usually in serious condition both physically and mentally, and the willingness of patients to take medicine is relatively strong, not only for antitumor drugs but also for analgesic drugs and sedative-hypnotic drugs. Another potential reason was that the adverse outcomes in older cancer patients were highly associated with PIM use, and the poor clinical outcome of cancer patients will further aggravate the prevalence of PIM use (Mohamed et al., 2020; Chen et al., 2021).

In our research, the most frequent PIM in Chinese older cancer outpatients was estazolam, according to the three criteria. Sleep disorder is common with advancing age and affects 36%–70% of older adults (Hishikawa et al., 2017; Patel et al., 2018), and it is further aggravated in older cancer patients. Consequently, estazolam is a benzodiazepine frequently used by older Chinese cancer patients. However, benzodiazepines are also linked to risks of mortality, falls, fractures, and depression among older adults (Kripke et al., 2002; Stone et al., 2008; Yaffe et al., 2014). Therefore, the risk of this category of medication use should be further evaluated for older cancer patients.

According to the results of logistic regression analysis, PIM-associated factors were the same among the three sets of criteria; older cancer outpatients who were ≥80 years of age, had more diseases, had polypharmacy, and had an irrational use of drugs and those who had lung cancer were more likely to receive PIMs. Furthermore, compared with other identified factors, polypharmacy is the most strongly associated independent risk factor. Patients with polypharmacy had more than two to three times the risk of PIM use compared with patients with one to four medications. In this study, the polypharmacy of older cancer outpatients was 22.53%, which is slightly little lower than the result of our other study (Tian et al., 2021), and this was similar to the results of Hsu et al.'s study, in which polypharmacy prevalence was lower in those with than without a cancer history (Hsu et al., 2021). Older cancer patients with age more than 80 generally have worse health and more multimorbidity than the general cancer population of older adults, and they are more likely to be exposed to PIM use (Lai et al., 2018). Our study found that, with the increase in multimorbidity in Chinese older cancer patients, the risk of PIM use gradually increased. This phenomenon is similar to older Chinese patients with other chronic diseases in some studies (Li et al., 2021; Zhao et al., 2021). The growth was more obvious with the 2014 STOPP criteria, as the PIM use of these criteria was more affected by the disease. In addition, unreasonable prescribing carries a higher risk of PIM use in Chinese older cancer outpatients. However, with the increase in prescription expenditures for cancer patients, the prevalence of PIM gradually declined. This was because the high expenditure on cancer prescriptions was mostly due to the use of antitumor drugs. However, the three criteria rarely involve antitumor drugs. Among all cancer diseases, only lung cancer was associated with PIM use. One study showed that at least half of patients with lung cancer have comorbidities, which would increase the risk of PIM use (Pluchart et al., 2021). Through these results, we suggested reducing unnecessary medications and performing medication reconciliation carefully for older cancer outpatients with taking multiple medications from the doctor or the pharmacist. At the same time, the criteria could be more refined according to the risk factors, such as the formation of special criteria for the outpatients who were ≥80 years of age and older lung cancer patients. This will further improve the feasibility and accuracy of the criteria.

Several limitations should be noted in this study. It was an observational study conducted in China, which is likely to cause some deviations in the results. These results need to be further confirmed by multicenter clinical trials. Second, there are no follow-up data for these older cancer patients when investigating PIM use by electronic medical data, so the correlation between PIM use and further clinical outcomes is not known. Finally, the patients attending outpatients of tertiary hospitals were the main focus of the study, and cancer outpatients who were in nursing homes and communities were not evaluated.

## Conclusion

This study investigated the use of PIMs in older cancer outpatients with multimorbidity in Chengdu based on the 2017 Chinese, 2019 AGS/Beers, and 2014 STOPP criteria. The results showed that the prevalence of PIM use was high in Chinese older cancer outpatients; poor-to-moderate concordance among the three criteria was observed; and age ≥80, more diseases, polypharmacy, irrational use of drugs, and lung cancer were risk factors for PIM use.

## Data Availability

The raw data supporting the conclusion of this article will be made available by the authors without undue reservation.
